# Commentary to ‘Incidence of vestibular schwannoma in Finland, 1990–2017’: Pediatric vestibular schwannomas: an overlooked epidemiological aspect?

**DOI:** 10.2340/1651-226X.2024.40652

**Published:** 2024-06-20

**Authors:** Gabriele Gaggero

**Affiliations:** Pathology Unit, IRCCS Istituto Giannina Gaslini, Genoa, Italy

**Keywords:** Vestibular schwannoma, pediatric, acoustic neurinoma, NF2

To the editor

I read with great interest the recent publication by Iivanainen A. et al. [1], which delves into the complicated epidemiological landscape of vestibular schwannomas (VS), also known as acoustic neurinomas. The authors’ meticulous analysis of Finnish national data sheds light on incidence trends and provides valuable insights into comparative aspects with existing literature data [1].

However, in the context of the comprehensive discussion, I believe it is important to address a crucial aspect that seems to have been somewhat overlooked – the consideration of pediatric VS.

While the incidence of VS in adults is well documented, ranging from 0.6 to 1.9 per 100,000 persons per year, it’s noteworthy that these tumors are significantly less common in the pediatric population [2–4].

In pediatric cohorts, VS often present as bilateral lesions in the context of neurofibromatosis type 2 (NF2), accompanied by other characteristic lesions such as other schwannomas, meningiomas, and gliomas [4, 5]. In contrast, sporadic cases without NF2 features or familial predisposition are extremely rare in children [4, 5–8].

Importantly, the existing literature highlights significant differences between adult and pediatric VS in terms of tumor biology, clinical presentation, and treatment response. In particular, pediatric VS exhibit different molecular pathogenetic substrates, vascularity, speed of growth of the tumors, and clinical outcomes compared to their adult counterparts. It’s critical to recognize these differences as they are likely to impact diagnostic and therapeutic strategies [9–12].

While adult VS are often amenable to standard treatment modalities, including surgical resection and stereotactic radiosurgery, the management of pediatric VS presents unique challenges due to their rarity and distinct clinical behavior. Furthermore, the long-term outcomes and prognostic factors associated with pediatric VS remain poorly understood due to the paucity of robust data and the predominance of case reports and small case series in the literature although some data indicate a more aggressive attitude, in terms of both post-surgical regrowth and recurrence [4, 12].

As a neuropathologist specializing in pediatric neuropathology, I feel compelled to emphasize the importance of delineating pediatric VS as a distinct entity from its adult counterpart. Although histologically overlapping, pediatric and adult VS represent fundamentally different pathologies with different clinical implications. Neglecting this conceptual distinction could lead to misconceptions in epidemiologic analyses and hinder our understanding of the true epidemiologic landscape of VS.

However, it should be emphasized that the rarity of pediatric VS, even if included separately from adult VS, would not, in my opinion, have significantly altered the statistical analysis performed by the authors, whose results remain robust and provide valuable insights into the overall epidemiology of VS.

In conclusion, I appreciate the authors for their careful investigation of the epidemiology of VS in the Finnish population, even if I suggest a more nuanced approach that includes the pediatric population as a separate age group. This approach would allow us to recognize the unique features and clinical courses of pediatric VS, thereby advancing our understanding of this complex entity.
