# Supplementary material for: Commentary to ‘Incidence of vestibular schwannoma in Finland, 1990–2017’: Pediatric vestibular schwannomas: an overlooked epidemiological aspect?
Source: Acta Oncol. 2024 Jun 20;63:40652. doi: 10.2340/1651-226X.2024.40652 (PMC11332545; doi:10.2340/1651-226X.2024.40652)
Supplement: Related texts

# Reply to the Commentary to ‘Incidence of vestibular schwannoma in Finland, 1990–2017’

**Authors:** Anssi Auvinen, Jani Raitanen

**Affiliations:** Tampere University, Faculty of Health Sciences, Tampere, Finland

We thank Dr Gaggero for his comments. Pediatric VS and its clinical characteristics constitute certainly an important topic worthy of a separate analysis. Our ability to address it is limited by the material. In our comprehensive case roster covering a pediatric population of some 88,000 children under the age of 15, we had only a total of seven cases during the 28-year study period (none at ages 0–4 years, 2 at ages 5–9 and 5 at ages 10–14, six in girls and one among boys). This corresponds to an incidence rate of 0.3 per million per year (95% CI: 0.1–0.6 per million). The sparse data did not lend itself to further analysis.
